# Safety Evaluation of Individual Pillboxes to Control Cross-Contamination in the Drug Circuit in Hospitals

**DOI:** 10.3390/ijerph16203878

**Published:** 2019-10-13

**Authors:** Claude Dussart, Caroline Boulliat, Isabelle Camal, Denis Bourgeois, Florence Carrouel

**Affiliations:** 1 Laboratory “Systemic Health Care”, EA4129, University Claude Bernard Lyon 1, University of Lyon, 69008 Lyon, France; denis.bourgeois69@orange.fr (D.B.); florence.carrouel@univ-lyon1.fr (F.C.); 2 Pharmacy Department, Military Teaching Hospital Desgenettes, 69003 Lyon, France; caroline.boulliat@intradef.gouv.fr (C.B.); pui.hiad@gmail.com (I.C.)

**Keywords:** pillbox, cross-contamination, drug circuit, bacteriological tests, medication, hospital

## Abstract

This study aims to evaluate the potential role of pillboxes used for the preparation and delivery of individual daily medical treatments in the drug circuit of the Military Instruction Hospital (France) as reservoirs of bacterial contaminants. Samples were obtained from 32 pillboxes after decontamination (T1), after preparation in the pharmacy (T2), after use in two different medical units (T3), and again after usual mechanical washing (T4). Qualitative (identification and antibiotic susceptibility) and quantitative (contamination rate and number of colony forming units—CFUs) bacteriological tests were performed. Susceptible and resistant strains of environmental saprophytes were identified. The pillbox contamination rate was relatively low at T1 (13%). It was significantly increased at T2 (63%, *p* = 0.001 vs. T1), again at T3 (88%, *p* < 0.05 vs. T2, *p* < 0.001 vs. T1), and finally decreased dramatically at T4 (31%, *p* < 0.001 vs. T3, *p* > 0.05 vs. T1). The number of CFUs was significantly increased at T2 compared with that of T1 (36.7 ± 13.4 and 5.36 ± 3.64, respectively, *p* < 0.001) and again at T3 (84.4 ± 19.4, *p* < 0.001 vs. T1 and T2) and was significantly reduced at T4 (7.0 ± 2.0 vs. T3, *p* < 0.001) to a level that was not significantly different from that at T1. So, the use of pillboxes to deliver individual medications to patients in the hospital is a potential risk factor for bacterial cross-contamination.

## 1. Introduction

Infection prevention and control (IPC) is a universally relevant component of all health systems and affects the health and safety of both people who use health services and those who provide them [1]. IPC is influenced by hospital hygiene and the expertise of the infection preventionists, pharmacists, and biologists for the prevention and management of the infection and infectious events [2].

The transversal and interdisciplinary process called the drug circuit consists of the prescription, dispensing, administration and therapeutic follow-up stages and includes the processing of information [3,4]. Within the hospital, the delivery of medicines takes the form of a global distribution, with nominative, computerized and centralized prescription preparation [5]. However, the proportion of potentially preventable nosocomial infections under routine working conditions remains unclear [6]. Additional precautions are needed for diseases transmitted by air, droplets and direct contact. These are termed “additional (transmission-based) precautions” and include the main hygiene standard such as hand hygiene, gown, gloves, surgical mask, goggles/face shield, and room placement, as well as the appropriate environmental measures [7].

The safety of taking medication at the hospital is based, among other things, on the intake of unit doses in pillboxes by identified patients prepared by the department’s pharmacy. A pillbox is defined as “any device, for an identified patient, according to the wording of a prescription, over a given period (day, week, month), a division of drug doses to be administered, according to the times indicated in decision the prescription (morning, noon, evening, night)” [8]. In terms of security, it is important to choose a pillbox with an efficient closing system and only one possible opening direction, which limits the risk of mixing treatments during transport or in case of pillbox fall.

The pillbox is an important object in the hospital. Used every day, the pillbox participates in the rhythm of the day for a patient. The unitary presentation of drugs necessary for the individual daily dispensing system entails less risk for the patient and is often more efficient for the establishment. It is also a link between the patient and the nurses. It strengthens the quality of care and medication adherence, secures the medical prescription and alleviates the workload of staff [9].

The drug circuit carries potential risks of contamination for the patient, related to the many stakeholders involved throughout the course of treatment [10]. Although the prevention of nosocomial infections is a major goal in healthcare facilities, the potential of the pillbox as a reservoir of nosocomial pathogens in hospital environments has never been investigated in the scientific literature. PubMed, Embase, and the Cochrane Library were searched in English with an end date of September 2018 using the Medical Subject Headings (MeSH) term “pillbox” and 12 different search terms for “drug circuit”: unit dose, unit of use pack, pill organizer, medication packaging, medication container, pill container, pill box, pillbox, blister pack, pill pack, special packaging AND medication. No study providing complete information was identified. Clear research gaps emerged.

This study is part of an evaluation of iatrogenic drug risk, securing the drug circuit and promoting the safety and quality of medication management. The aim of this research was, therefore, to quantitatively evaluate potential pillboxes cross-contamination events along their medication dispensation circuit in a French Military Instruction Hospital. The second objective was to identify the potential source of contamination and to evaluate the cleaning protocol.

## 2. Materials and Methods

### 2.1. Location and Type of Study

The Military Instruction Hospital Desgenettes (MIH) is a hospital with 148 beds (including 22 days hospital beds) located in Lyon, France. Its primary mission is to ensure the health support of the armed forces in all of southeast France. Nonetheless, it is open to all other insured persons and participates in the public hospital service. Observations were conducted from June through July 2014 in the two following inpatient units within MIH:

- A medical intensive care unit (ICU), surgical ICU, and general medical/surgical unit of dermatology in a 14-bed adult tertiary care hospital,
- A medical/surgical ICU and general medical/surgical unit of an 18-bed orthopaedic acute care hospital.

In this observational study, we described four levels of potential contamination rates of pillboxes inside the drug circuit in two successive sets of experiments. The MIH Institutional Review Board approved the study procedures.

### 2.2. Definition

The drugs were presented in dry oral forms (tablet, capsule, “small” bag of powder) and transdermal forms (“small” patch). Depending on the size of the pillbox and the available space, it was possible to add other forms of medication.

Pillboxes used at MIH and selected for study were daily pill organizers with four a.m./p.m. compartments and a large medication dispenser case for medicines (Distri 1 Eurojour^®^, Cima, Ponts, France).

### 2.3. Procedure

This study was an observational study. During a one-day period, study investigators collected data on the same pillbox. Each observer conducted 20 h of observations between the hours of 5:00 and 23:30 from the medication cabinet to the wash service. Pillboxes were randomly selected from all the medical prescriptions received the previous night before midnight in the pharmaceutical service. Using a standardized form for each pillbox, investigators recorded the date, start, and end time of the observation period. Observers classified each pillbox entering the drug distribution circuit into one of the following four periods: (i) after decontamination (T1 sample); (ii) after use for medicine preparation in the pharmacy (T2 sample); (iii) upon return from the medical unit (T3 sample) and (iv) after washing in the dishwasher (T4 sample).

### 2.4. Professionals Involved in the Dispensing of Medication

The dispensing of medication to the patient is the act under the direct responsibility of the pharmacist. The staff involved in the pillbox circuit are respectively: (i) The pharmacy preparer who, under the supervision of a pharmacist, identifies pillboxes and prepares medicines, (ii) the non-clinical staff from the pharmaceutical team delivers pillboxes delivered to medical unit, and (iii) the graduate nurse (GN) who prepares the doses to be administered extemporaneously and administers the drugs to the patient.

### 2.5. Pillbox Preparation and Use

Pillboxes were used in the pharmacy to prepare daily individual treatment according to the medical prescription with single daily medication. The pillboxes were then delivered to medical units by non-clinical staff. The drug was administered 3–4 times a day according to the prescription of the doctor by the nursing staff without the patient contacting his or her pillbox. Boxes were returned to the pharmacy the next day where they were washed in a specific dishwasher.

Since sterilization was not possible in HIA, boxes were submitted to strict decontamination. The few organisms identified were probably caused by the environment since the sampling took place in the usual washing room where several personnel were working. The manipulator managed to avoid direct contact with the box by wearing sterile gloves and a mask. In the Hospital Pharmacy Unit, the pharmacy preparers performed the activity of individual nominative dispensing manually for 120 hospital beds. A pharmacist controlled all pillboxes.

Drug preparation was linked to general ergonomic specificities: (i) Planning time for drug preparation, (ii) informing colleagues to avoid disturbances, (iii) decontaminating the work surface and the medication tray with a surface decontaminant, and (iv) making sure the pill dispensers were clean and using single-use buckets. Pillbox maintenance depends on the maintenance frequency and nature of immersion cleaning. Cleaning can be performed by hand, but the use of the dishwasher is preferred.

To evaluate the potential contamination source, the pillboxes used in this study were cleaned using a specific protocol [11]. Boxes were successively immersed for 15 min in a Desintex^®^ 0.25% solution (Rochex, Annemasse, France), rinsed with water, cleaned with the detergent Sopromod^®^ (Packing Dispatch, Saint-Alban-Leysse, France), rinsed again, immersed in a bleach solution (0.1% active chlorine) for 15 min and rinsed with sterile water. Boxes were finally dried using sterile tissue. As in the current practice, pillboxes were then stored in the pharmacy and used by pharmacy assistants to prepare daily medications for each patient according to the medical prescription.

In this study, 32 prescriptions were randomly selected for patients belonging to the dermatology and orthopaedic surgery units. Identified boxes were used to prepare these treatments and were delivered to the medical units. The next day, empty boxes were returned to the pharmacy and were, as in daily practice, washed using a dishwasher (Miele professional G7882 CD, Le Blanc-Mesnil, France) with Septoclean Neodisher^®^ detergents (Dr. Weigert France SAS, Roissy Charles De Gaulle, France) before being stored and used again for another patient.

### 2.6. Bacterial Contamination, Identification and Antibiotic Susceptibility

The collected samples from the whole external surface of the pillbox, Swab Rinse Kit (SRK environmental swab system, Copan, CML, Nemours, France) were used in accordance with the swab sampling technique [12], which is the recommended method to recover microorganisms from solid surfaces [13]. For each studied pillbox, one sterile swab was rubbed and rolled firmly across the sampling area. The sampling area from a pillbox is composed of five surfaces that are sampled one after the other but with the same swab. The swab was moved across the sampling area using parallel and close scrapes as follows: one stroke in horizontal direction, one stroke in vertical direction, one stroke in a diagonal direction, and one stroke in the other diagonal direction [14]. The swab was immersed in 2.5 mL of isotonic rinse solution (SRK environmental swab system).

In the first set of experiments, a quantitative measure of bacterial contamination was performed by inoculating 200 µL of the swab rinse solution to chocolate culture medium (Biomérieux, Marcy-l’Etoile, France). The number of colony forming units (CFUs) was measured after a 48-h culture period at 37 °C with carbon dioxide enrichment.

In another set of experiments, each sample was inoculated for 24 to 48 h at 37 °C with carbon dioxide enrichment in chocolate culture medium (Biomérieux). Colonies from positive cultures were then inoculated in COS blood-agar medium (Columbia agar + 5% sheep blood) (Biomérieux). After another 24 h, cultures were Gram stained and identified using an API (Analytical Profile Index) system (Biomérieux). Antibiotic susceptibility testing was performed using an agar diffusion test on a Müller-Hinton agar plate (Biomérieux) with specific antibiotic disks (Biorad, Marnes la coquette, France) and read with SirScan I2A (Montpellier, France).

Contamination rates were calculated by pooling data from both sets of experiments.

### 2.7. Assessment and Outcomes

All selected pillboxes were followed after the inclusion visit for 1 day.

#### 2.7.1. Experimental Protocol

**Figure 1:**
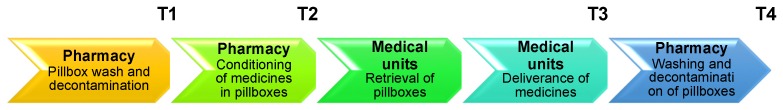
Workflow of the experiment. T1, T2, T3 and T4 represent the different sampling times. T1: after cleaning, T2: after use for medicine preparation in the pharmacy, T3: upon return from the medical unit, T4: after washing in the dishwasher.

As presented in Figure 1, boxes were sampled as follows:

- after decontamination (T1 sample);
- after use for medicine preparation in the pharmacy (T2 sample);
- upon return from the medical unit (T3 sample);
- after washing in the dishwasher (T4 sample).

Two sets of experiments were performed on two non-consecutive weeks.

#### 2.7.2. Outcomes

The primary efficacy endpoint was the contamination rate (%) with both susceptible and resistant bacterial strains at T3, and the secondary endpoints included the number of CFUs per box (mean ± s.e.m.) at T1, T2, and T4.

### 2.8. Statistical Analysis

McNemar’s tests were performed to compare contamination rates at different times; Fisher’s exact tests were used to compare contamination rates among different medical units. CFU data were statistically analysed using an on ranks one-way ANOVA (Kruskal-Wallis test) for repeated measures followed by all pairwise multiple comparisons with Student-Newman-Keuls test. All statistical tests were performed using Sigmastat 3.5 software (Jandel Scientific Software, San Jose, CA, USA). The significance level was set at *p* < 0.05.

## 3. Results

### 3.1. Bacterial Identification

Bacterial stains identified on pillboxes are listed in Table 1. They were mostly susceptible strains, but resistant strains were also detected.

### 3.2. Contamination Rates 

**Figure 2:**
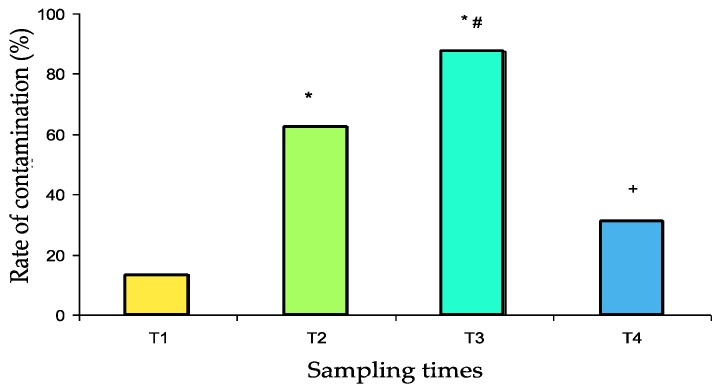
Contamination rate of 32 pillboxes with both susceptible and resistant bacterial strains at the different sampling times (T1 to T4). T1: after cleaning, T2: after use for medicine preparation in the pharmacy, T3: upon return from the medical unit, T4: after washing in the dishwasher. * *p* ≤ 0.001 vs. T1, # *p* < 0.05 vs. T2, + *p* < 0.001 vs. T3.

The contamination rates are presented in Figure 2. After scrupulous washing (T1 samples), the pillbox contamination rate was relatively low (13%). Contamination significantly increased after use in the pharmacy (T2 samples: 63%, *p* = 0.001 vs. T1) and again after use in the medical units (T3 samples: 88%, *p* < 0.05 vs. T2, *p* < 0.001 vs. T1). After washing in the dishwasher, the contamination rate decreased dramatically (T4 samples: 31%, *p* < 0.001 vs. T3) to a value that was not significantly different from baseline.

No significant difference was found between the contamination rate of boxes departing or returning from the two medical units studied (Table 2). Contamination rates with resistant strains were 0% at T1, 18.8% at T2, 18.8% at T3 and 6% at T4. No significant difference was observed.

### 3.3. Number of Colonies Contaminating the Pillboxes 

**Figure 3:**
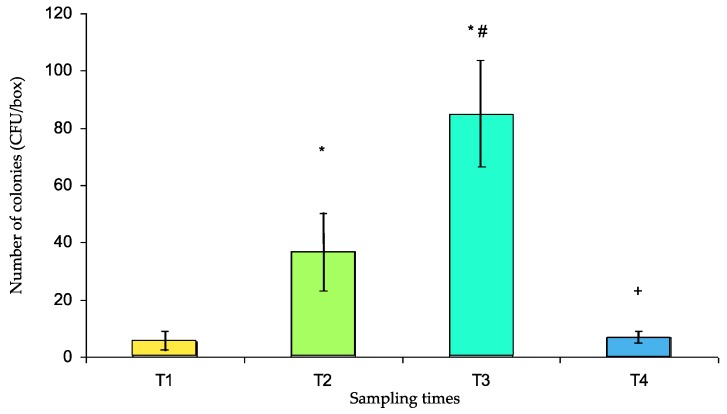
Number of colony forming units (CFUs) per box (mean ± s.e.m.) at different sampling times (T1 to T4). T1: after cleaning, T2: after use for medicine preparation in the pharmacy, T3: upon return from the medical unit, T4: after washing in the dishwasher. * *p* < 0.001 vs. T1, # *p* < 0.001 vs. T2, + *p* < 0.001 vs. T3.

The CFUs are presented in Figure 3. The number of CFUs significantly increased at T2 compared with that at T1 (36.7 ± 13.4 and 5.36 ± 3.64, respectively, *p* < 0.001), rose again at T3 (84.4 ± 19.4, *p* < 0.001 vs. T1 and T2) and was significantly reduced at T4 (7.0 ± 2.0 vs. T3, *p* < 0.001) to a level that was not significantly different from that at T1.

## 4. Discussion

Healthcare-associated infections (HAIs) pose a significant health care and cost burden [15]. HAIs affect millions of patients every year and are the most common complication of healthcare delivery globally [16]. There is evidence that a significant proportion of nosocomial infections could be avoided [17]. Despite control efforts, the burden of healthcare-associated infections in Europe is high and leads to approximately 37,000 deaths each year [18]. In France, although the prevalence of those infections has decreased in recent years, they still represent an additional direct medical cost of hundreds of millions of euros [19].

There also needs to be more scrutiny of the reasons for the failure of research implementation through an examination of the “soft periphery” that comprises the organizational structure, systems and people that are responsible for implementing and sustaining an intervention [20]. This study demonstrated that the use of pillboxes to deliver medication to patients could be a vector of bacteriological contamination under the usual conditions observed in the Military Hospital.

Bacteria identified on pillboxes after manipulation, both at the pharmacy and in medical units, were almost all environmental saprophytes that are common on human skin (*Staphylococcus epidermidis, S. warneri* and *S. hominis,* Corynebacterium and *Micrococcus*), on the human head (*Staphylococcus capitis*) or in the mouth (*Streptococcus sanguinis* and *S. oralis*). Box contamination may easily have been caused by manipulation with tainted hands or by coughing. The preparation and transport of a pillbox cannot be performed without essential rules of hygiene, such as washing of the hands, cleaning of the work surface, and using clean pillboxes. Before any manipulation of the treatments, the GN was washed with a hydroalcoholic solution. In France, this is an obligatory procedure imposed on all hospital caregivers by the Ministry of health. The GN ensured that the pillbox had been cleaned and checked the traceability on the chart of care. The hygiene of the pillboxes must be defined with a regular frequency within a procedure.

However, the contamination is worrying because these bacteria are involved in several infections. Previous studies have reported that coagulase-negative staphylococci (CoNS) isolates, such as *S. epidermidis* (26.2%), *S. haemolyticus* (25.4%), and *S. capitis* (17.2%), were frequently recovered from hospital environments (air, walls, floors and medical equipment) [21]. CoNS represent one of the major nosocomial pathogens, having a substantial impact on human life and health. *Staphylococcus epidermidis* is among the most frequent bacterial sources underlying bacteraemia and sepsis [19]. *Staphylococcus warneri* has been isolated in prosthetic device-related infections and has been reported as a rare cause of endocarditis [22,23,24,25,26]. *Staphylococcus hominis* is the third species of CoNS most frequently isolated from specimens of patients with hospital-acquired infections [27]. *Staphylococcus capitis* has the potential for both human disease and nosocomial spread [28].

Among the group of non-diphtheriae *corynebacteria*, a growing number of species have been found to be aetiological agents of human infections, mainly opportunistic and nosocomial infections [29]. *Corynebacterium striatum* is recognized as a potential pathogen of both immunocompromised and immunocompetent hosts [30,31]. It is reported to be a pathogen of osteomyelitis, arthritis, endocarditis, meningitis and cerebrospinal fluid-shunt infection [32,33,34,35,36]. *C. striatum* can also cause bloodstream infections in immunocompromised patients [37,38].

Concerning bacteria from the oral cavity, *S. sanguinis* was recognized as one of the top three causes of endocarditis, alongside two other genera, *Enterococci* and *Staphylococci* [39].

More unusual is the presence of *Leuconostoc*. This organism is commonly used in the dairy, wine, and sugar industries. It has also been identified as a potential pathogen, especially in immunocompromised patients, and can cause nosocomial outbreaks. Although most strains identified are not pathogenic for humans, these bacteria may cause infections in patients whose immune system is compromised. Resistant strains were identified in only 11% of samples. However, the eventual contamination of patients with these organisms could be difficult to manage. Therefore, it is highly important to avoid contamination of patients by direct contact with the box. The criteria for choosing the model must consider (i) the typology of patients, (ii) type of hospitalization and average length of stay, as well as (iii) transport modalities (robustness, ease of maintenance) and (iv) ergonomics: identifiable and modular pillboxes, presented in eight pillboxes daily.

The nominative dispensing of drugs is part of the overall quality of the care development strategy at the hospital. Pillboxes, as an environmental vehicle for bacterial contamination, are used for unit dose conditioning (full, half and quarter dose) of all non-identifiable drugs. It is a good way to reduce iatrogenesis during administration and to reduce the contamination of dry, oral forms [40]. The results of this study allowed us to keep workers informed about nosocomial infection and hygiene.

Transport in the care units remains improvable. Except in the rare cases of dispensing by name, the staff of the pharmacy no longer physically control the drugs as soon as they cross the door of the pharmacy. Transportation to the wards is handled by the hospital’s service staff or by the health care staff who come to pick up their order. The use of pillboxes as a part of infection control requires a procedure. Choosing an easily cleanable pillbox is a priority, however, the term “easily cleanable” needs to be defined, especially in terms of what kind of cleaning. Our research hypothesis involved the use of sterilized pillboxes, which, upon reading the literature, did not correspond to the practices. Decontamination is best performed with a thermo-washer with frequencies that are not regulated.

In our study, the contamination rate significantly increased after manipulation in the pharmacy. At the same time, the number of CFUs also increased significantly. The contamination rate and number of CFUs rose again significantly after manipulation in the medical units. These results indicated that contamination increased with the time and number of persons manipulating the box, leading to almost complete contamination. Finally, the usual washing protocol performed was able to significantly reduce the contamination and bring it back to its baseline value. However, none of the bacterial strains present in patients (several presenting infected skin wounds) were found on the box, indicating the absence of cross-contamination by patient manipulation of boxes.

Our study has the following strengths. The quality of the evidence of the contamination of the inanimate environment was judged according to whether the following four factors had been measured: (1) the degree of contamination of the nosocomial environment by specific pathogens, (2) temporality (i.e., whether the environment was contaminated before or after pillbox use), (3) the assessment of the quality of cleaning of fomites, and (4) cleaning improvement after the stockage of pillboxes for other interventions for the reduced risk of cross-contamination.

Our study has the following limitations. This study and its resulting interpretations, in the absence of published references, should be considered as a pilot survey with all the resulting objections because of the number of arms, the limited number of pillboxes, and the inclusion of a unique hospital site.

It is possible, for instance, that staff may have altered their behaviour from normal practices during unit observations. The focus of this study was on two specific clinical units in the hospital. Otherwise, it is not known to what extent surfaces, including pillboxes, must be cleaned in order to prevent cross-transmission; acceptable residual bioburden levels were not established. This makes the evaluation of cleaning technologies difficult to standardize.

These results demonstrate that the use of pillboxes to deliver individual medications to patients in the hospital causes a potential risk for bacterial cross-contamination. A standard procedure for cleaning and disinfection of reusable equipment, including staff training, should be provided inside the MHA. It appears useful to communicate on the measures of hygiene to be respected during the preparation of the pillboxes shall be remembered: washing of the hands, cleaning of the work plan, use of decontaminate pillboxes. Failure to comply with these measures and deconditioning of dry oral forms explain why contamination of dry oral forms can reach 37% of doses [41]. Additionally, the use of sterilization or the use of single-use pillboxes should be investigated.

This study cannot validate one hundred percent of the effectiveness of the washing protocol usually performed at the hospital. There is no single ideal disinfectant [42]. It is important to keep pharmacy personnel and medical caregivers informed of the potential contamination by manipulating boxes. This training is inseparable from high-priority confounding factors such as hand hygiene, compliance procedures, and surface disinfection.

Certainly, the pillbox is a neglected risk. In accordance with the rules, the level of disinfection/cleaning required is classified as non-critical. The risk level is low [43]. Our study demonstrates that most boxes are clean but not sterile. However, its use follows manipulation procedures and a specific circuit within the hospital; it is used several times a day for the same patient and the pillbox is stored on a cart common to the patients in the staff room between each distribution (morning, noon, evening, night).

Finally, the distribution of drugs concerns all patients at the same time. Even if the pillbox of a patient never enters the room of another patient, there is nevertheless a risk of additional contamination related to contact by the medical staff and airborne contamination during the distribution circuit.

One easy way to reduce the potential risk of pathogen transmission could be to use only one unique pillbox for each patient. Purchasing a single use pillbox that is supplied in sterile packaging can help reduce the risk of contamination. Using devices that are single-use only can greatly decrease the risk of cross-contamination, but proper sterilization techniques must still be practised. New guidelines focused on pillboxes may have a place in infection prevention programmes as part of a multimodal approach, assuming that sufficient resources exist to ensure the basic components of the improvement strategy are in place.

A suggestion for future research is to develop methodologically sound studies using control cases, a high number of services, and complete reporting of bacteriological information at each stage of the process in order to clarify the effects of the manipulation of pillboxes on the quality assurance of hospital drug distribution.

## 5. Conclusions

The use of pillboxes to deliver individual medications to patients in the hospital is a potential risk factor for bacterial cross-contamination. However, our results indicate that this risk is low even if it is present. So, the classical circuit of preparation and use of pillboxes must be scrupulously respected.

## Figures and Tables

**Table 1:** Bacterial strains identified from 32 pillbox samples upon return from medical units.

Bacteria	Total *n* (%)	Resistant Strains *n* (%)
*Staphylococcus capitis*	13 (40.6)	
*Staphylococcus warneri*	6 (18.8)	
*Staphylococcus epidermidis*	6 (18.8)	4 (12.6)
*Staphylococcus hominis*	2 (6.2)	2 (6.2)
*Corynebacterium striatum*	1 (3.2)	
*Leuconostoc* sp.	1 (3.2)	
*Micrococcus lylae*	1 (3.2)	
*Streptococcus sanguinis*	1 (3.2)	1 (6.2)
*Streptococcus oralis*	1 (3.2)	

*n*: number of positive samples.

**Table 2:** Contamination rates with both resistant and sensitive bacterial strains at the different sampling times (T1 to T4) for the medical units of dermatology (*n* = 14) and orthopaedic surgery (*n* = 18).

Bacteria	Dermatology *n* (%)	Orthopaedic Surgery *n* (%)
T1	2 (15.4)	2 (11.8)
T2	10 (71.4)	10 (55.6)
T3	11 (78.6)	17 (94.4)
T4	3 (21.4)	7 (38.9)

*n*: number of positive samples, T1: after cleaning, T2: after use for medicine preparation in the pharmacy, T3: upon return from the medical unit, T4: after washing in the dishwasher.
